# Infiltrating macrophages replace Kupffer cells and play diverse roles in severe alcohol-associated hepatitis

**DOI:** 10.1038/s41423-025-01343-1

**Published:** 2025-09-17

**Authors:** Yang Wang, Yukun Guan, Dechun Feng, Luca Maccioni, Maria A. Parra, Brandon Peiffer, Bryan Mackowiak, Takashige Kuwabara, Kiyoshi Mori, Masashi Mukoyama, Ramon Bataller, Zhaoli Sun, Bin Gao

**Affiliations:** 1 Laboratory of Liver Diseases, National Institute on Alcohol Abuse and Alcoholism, NIH, Bethesda, MD USA; 2 Department of Surgery, Johns Hopkins University School of Medicine, Baltimore, MD USA; 3 Department of Nephrology, Kumamoto University Graduate School of Medical Sciences, Kumamoto, Japan; 4 Department of Nephrology and Kidney Research, Shizuoka General Hospital, Shizuoka, Japan; 5 School of Pharmaceutical Sciences, University of Shizuoka, Shizuoka, Japan; 6 Graduate School of Public Health, Shizuoka Graduate University of Public Health, Shizuoka, Japan; 7 Liver Unit, Hospital Clinic, Barcelona, Spain; 8 Steatohepatitis and Liver Transplantation, Institut d’Investigacions Biomèdiques August Pi i Sunyer (IDIBAPS), Barcelona, Spain; 9 Mechanisms of liver injury, cirrhosis progression and liver transplantation, Centro de Investigación Biomédica en Red Enfermedades Hepáticas y Digestivas, Madrid, Spain

**Keywords:** C1Q, APOE, S100A8, Macrophages, Neutrophil apoptosis, Immunological disorders, Cell biology

## Abstract

Patients with alcohol-associated cirrhosis (AC) may develop severe alcohol-associated hepatitis (sAH), a disease with high short-term mortality. Our previous studies demonstrated that sAH, but not AC livers, are infiltrated with a high number of self-sustaining IL-8^+^ neutrophils that likely drive the transition from AC to sAH. Monocyte-derived macrophages (MoMFs) also infiltrate the liver in sAH, but their roles remain largely obscure. In the present study, we characterized liver macrophages in human liver explants from sAH and AC patients. Our data revealed a marked reduction in Kupffer cells, whereas MoMFs were increased in sAH and AC. Single-cell RNA-Seq analyses revealed several populations in both AC and sAH, including *C1Q*^+^, *S100A8*^*+*^*, APOE*^+^, *TNF*^*+*^ and *VSIG4*^*+*^ macrophages, with sAH containing unique *C1Q*^*+*^ macrophages potentially playing a role in removing apoptotic neutrophils in sAH. *C1Q*^*+*^ macrophages also express many genes involved in phagocytosis and proinflammatory and anti-inflammatory functions, suggesting that *C1Q*^*+*^ macrophages have diverse functions in sAH. The roles of *C1Q*, *S100A8*, and *APOE* were further examined in experimental models of alcohol-induced liver injury. Our data revealed that *C1q* KO mice and macrophage-specific *S100a8* KO mice presented similar alcohol-induced liver injury and hepatic neutrophil infiltration, while *Apoe* KO mice developed much more severe liver injury than did WT mice following chronic-plus-binge ethanol challenge. Taken together, sAH and AC are infiltrated with multiple populations of macrophages that perform diverse functions to drive chronic disease progression. Unique *C1Q*^+^ macrophages in sAH play a compensatory role in removing dead cells but may also promote inflammation in sAH.

## Introduction

Alcohol-associated liver disease (ALD) is one of the most common chronic liver diseases worldwide, encompassing a spectrum of conditions ranging from simple steatosis to steatohepatitis, cirrhosis, and hepatocellular carcinoma (HCC) [1]. Patients with chronic ALD who engage in excessive alcohol consumption may develop alcohol-associated hepatitis (AH), which presents with severe clinical manifestations such as jaundice and complications of portal hypertension. Severe AH (sAH) is associated with high short-term mortality, and currently, early liver transplantation remains the only effective curative treatment [2]. Given that sAH is characterized by significant liver inflammation, targeting inflammatory pathways has been a major focus of both preclinical and clinical research [3, 4]. Several clinical trials have attempted to treat sAH by targeting inflammatory mediators such as TNF-α or IL-1β, but these efforts have largely failed [5, 6]. The reasons for these failures may include the limitation of targeting single inflammatory mediators rather than addressing the complex and multifaceted inflammatory networks involved in sAH [7] or the possibility that the primary driver of inflammation in sAH has yet to be identified. Future research should focus on elucidating the underlying mechanisms of sAH-related inflammation and developing more comprehensive and effective therapeutic strategies.

Using multiplex immunofluorescence staining analysis, we recently identified substantial infiltration of inflammatory cells, predominantly consisting of macrophages, neutrophils, and T cells, in sAH livers [8]. Furthermore, single-cell RNA sequencing revealed a significant population of self-sustaining interleukin-8 (IL-8)^+^ neutrophils, which were highly abundant in sAH livers but present at much lower levels in peripheral blood neutrophils from sAH patients and those with alcohol-associated cirrhosis [9]. IL-8 is a key chemokine that not only promotes neutrophil chemotaxis but also enhances neutrophil activation [10]. On the basis of these findings, we propose that IL-8^+^ neutrophils play a central role in driving the vicious cycle of inflammation in sAH and represent a promising therapeutic target for AH [11]. Therefore, clinical trials targeting IL-8 signaling for sAH treatment are warranted.

Elevated macrophage infiltration is also observed in the livers of sAH patients; however, their specific roles in sAH pathogenesis remain largely unknown [8]. To address this knowledge gap in an unbiased manner, we applied single-cell RNA sequencing to delineate the macrophage landscape in both the liver and peripheral blood of sAH patients. This analysis identified several distinct macrophage populations in sAH livers characterized by the expression of genes involved in inflammation, phagocytosis, and liver repair. To further investigate the functional significance of key dysregulated genes, we employed multiple experimental models of alcohol-induced liver injury with various genetically modified mouse lines. Overall, our research provides a comprehensive understanding of macrophage dynamics in sAH and lays the foundation for the development of macrophage-based therapeutic strategies for AH patients.

## Results

### Kupffer cells are replaced by infiltrating monocyte-derived macrophages in sAH

**Fig. 1:**
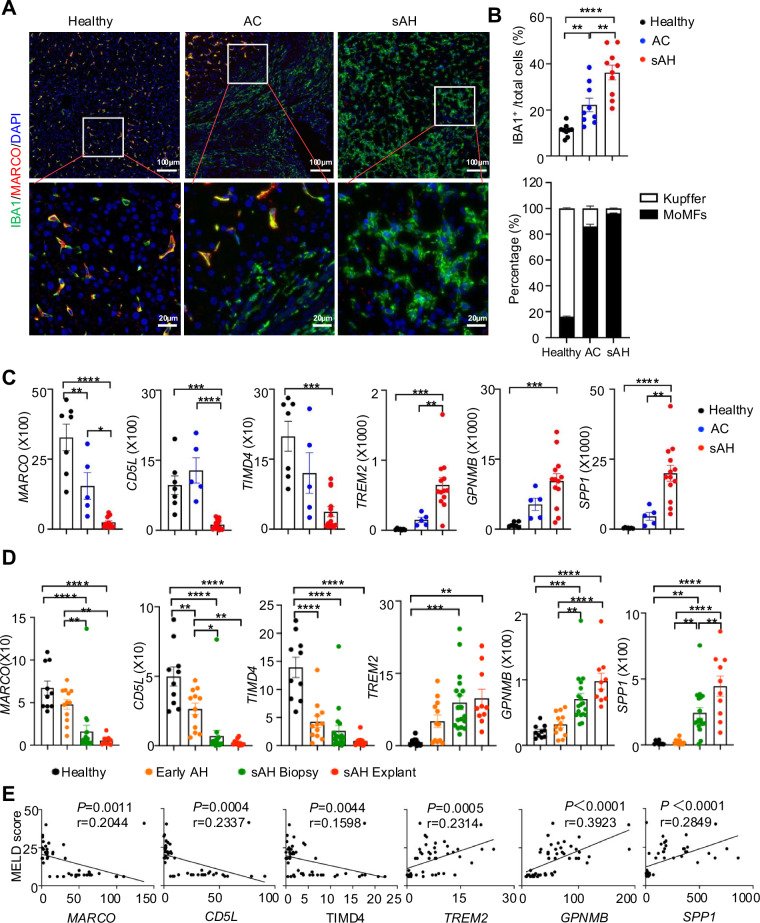
Kupffer cells are replaced by infiltrating macrophages in sAH. **A** Liver tissues from healthy controls (*n* = 9), alcohol-associated cirrhosis (AC) patients (*n* = 9), and alcohol-associated hepatitis (sAH) patients (*n* = 10) were analyzed via multiplex immunofluorescence staining for IBA1 (green) and MARCO (red). Representative immunofluorescence images are shown. Upper panel scale bars, 100 µm. Lower panel scale bars, 20 µm. **B** Quantification of IBA1⁺ Kupffer cells and macrophages as a proportion of total liver cells is shown (upper). The percentages of Kupffer cells and infiltrating macrophages among total IBA1⁺ cells were also quantified (lower). **C** Hepatic expression levels of Kupffer cell markers (*MARCO, CD5L*, and *TIMD4*) and infiltrating macrophage markers (*TREM2, GPNMB*, and *SPP1*) were analyzed via RNA-seq data from healthy controls (*n* = 7), AC patients (*n* = 5), and sAH patients (*n* = 13). Gene expression levels are presented as relative read counts. **D** Hepatic expression of Kupffer cell and infiltrating macrophage markers was analyzed via RNA-seq data (phs001807.v1. p1) from healthy controls (*n* = 10), early AHs (*n* = 12), sAH biopsies (*n* = 18), and sAH explants (*n* = 10). Gene expression levels are presented as transcripts per million. **E** Correlation analyses between MELD scores and Kupffer or infiltrating macrophage markers from RNA-seq data (phs001807.v1. p1) were performed. The data are presented as the means ± SEMs. Statistical significance was determined via one-way ANOVA followed by Tukey’s post hoc test for multiple comparisons. *P < 0.05, **P < 0.01, ***P < 0.001

To characterize liver macrophages in sAH and AC, we performed multiple staining for ionized calcium-binding adapter molecule 1 (IBA1), cluster of differentiation 68 (CD68), T-cell immunoglobulin and mucin domain-containing protein 4 (TIM4) and macrophage receptor with collagenous structure (MARCO) on human liver tissues from healthy controls, AC, and sAH. IBA1 and CD68 serve as panmacrophage markers, whereas MARCO and TIM4 are specifically expressed by liver-resident Kupffer cells [12, 13]. TIM4 and MARCO staining overlapped well in the liver tissues we stained (Fig. S1A). IBA1 and CD68 staining also overlapped, except in AC fibrotic regions where the macrophages were strongly stained with IBA1 but not with CD68, suggesting that IBA1 is a broader panmacrophage marker than CD68 is (Fig. S1A). Therefore, we choose IBA1 and MARCO for the following experiments. Notably, the percentage of IBA1⁺ macrophages in total liver cells increased from approximately 10% in healthy controls to ~40% in sAH patients and ~30% in AC patients (Fig. 1A, B). Moreover, in healthy controls, the majority (80–90%) of macrophages were Kupffer cells coexpressing IBA1 and MARCO (Fig. 1A, B, and S1A, B). In contrast, ~90% of the macrophages in sAH patients are infiltrating monocyte-derived macrophages (MoMFs), which expressed IBA1 but not MARCO (Fig. 1A, B, and S1A, B), indicating that Kupffer cells are largely replaced by MoMFs. In AC patients, Kupffer cells were preserved only within certain hepatocyte nodules, whereas all macrophages in fibrotic regions were MoMFs (Fig. 1A).

To investigate whether gene expression patterns of Kupffer cell and MoMF markers could distinguish sAH patients from healthy controls, we performed bulk RNA-seq and analyzed the expression of Kupffer cell markers and MoMF markers [14]. The Kupffer cell markers *MARCO, CD5L*, and *TIMD4* were significantly downregulated in AC and sAH patients compared with healthy controls, whereas the MoMF markers *TREM2*, *GPNMB*, and *SPP1* were upregulated in AC and sAH patients (Fig. 1C).

To validate these findings, we analyzed bulk RNA-seq data from another cohort, including livers from healthy controls and early AH, sAH (biopsy), and sAH (explant) patients [15]. In this dataset, Kupffer cell markers were also markedly reduced, whereas MoMF markers were significantly elevated in both biopsy and explant sAH samples (Fig. 1D). Additionally, *MARCO, CD5L, GPNMB*, and *SPP1* could further differentiate early AHs from sAH patients (Fig. 1D).

We then examined whether the expression of Kupffer cell and MoMF markers was associated with liver injury by correlating their expression with MELD scores. As shown in Fig. 1E, Kupffer cell marker expression was negatively correlated with the MELD score, whereas MoMF marker expression was positively correlated with the MELD score.

Finally, we assessed whether these markers could distinguish healthy controls from patients with liver diseases of other etiologies. Interestingly, our data revealed that these markers were not effective in differentiating healthy controls from patients with other etiology-associated liver diseases (Fig. S1C, D).

### scRNA-seq identifies inflammatory and phagocytic *C1Q*^*+*^ macrophages in the livers of patients with sAH

**Fig. 2:**
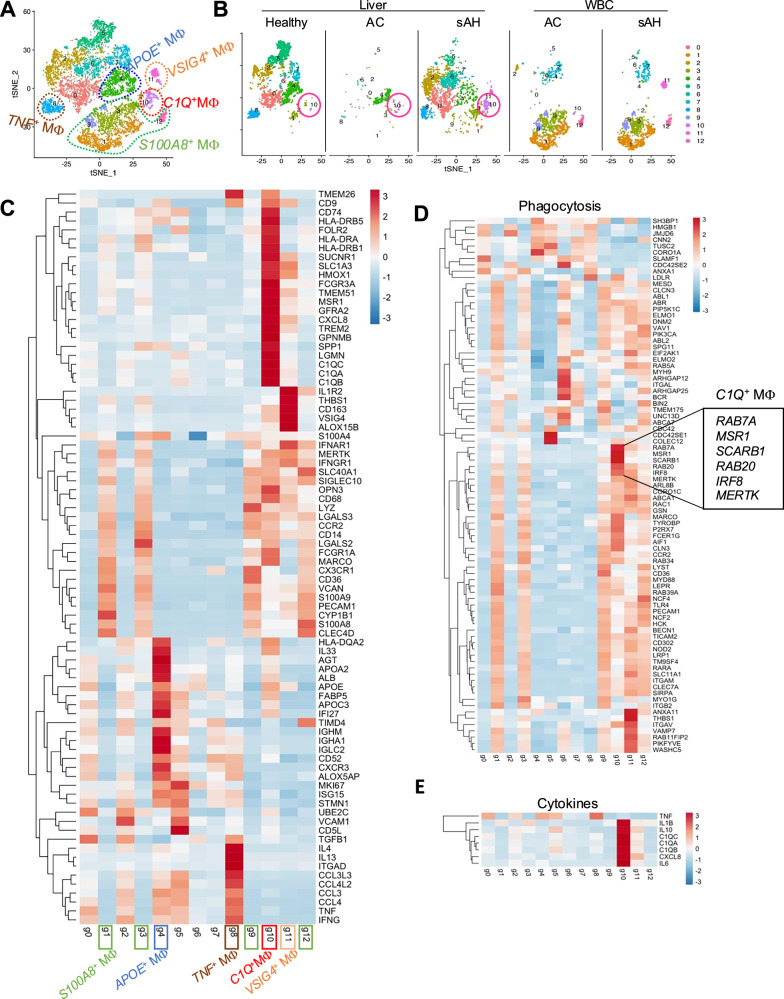
scRNA-seq reveals macrophage heterogeneity in sAH. **A** Single-cell RNA sequencing analysis was performed on liver cells from 5 healthy controls, 3 AC patients, and 5 sAH patients, as well as peripheral white blood cells (WBCs) from 3 AC patients and 4 sAH patients. t-SNE plots showing the clustering of all the macrophage populations. **B** t-SNE plots showing macrophage clusters separated by patient group. **C** Heatmap illustrating the key signature genes of each macrophage subtype. **D** Heatmap displaying the expression of phagocytosis-related genes across macrophage subtypes. (**E**) Heatmap showing the expression of cytokine genes among all the macrophage subtypes

We previously performed single-cell RNA sequencing (scRNA-seq) analysis of liver and peripheral white blood cells (WBCs) from healthy controls and AC and sAH patients to characterize the immune landscape [9]. We identified self-sustaining IL8^+^ neutrophil infiltration in the livers of sAH patients as a driver of inflammation [9], but the macrophages identified from these scRNA-seq data were not analyzed. To explore the heterogeneity of macrophages in the livers and peripheral blood of sAH patients, we analyzed scRNA-seq data and identified 13 subtypes of macrophages (Fig. 2A, B).

We then classified major functional macrophage phenotypes by evaluating the expression of cell type-specific markers and differentially expressed genes (DEGs) (Figs. 2C, S2A). These included *C1Q*^*+*^ macrophages (*C1qA, C1qB, C1qC, CD68, FCGR3A, MARCO, FOLR2, SLC40A1, HMOX1, SUCNR1, TMEM26, CD74, LGMN, HLA-DRA, HLA-DRB5, and HLA-DRB1*), *S100A8*^*+*^ macrophages (*S100A8, S100A9, CX3CR1, CCR2, CD14, LYZ, VCAN, CD36, PECAM1, and SIGLEC10*), *APOE*^*+*^ macrophages (*APOE, ALOX5AP, FABP5, APOA2, ISG15, S100A4, CD52, IL33, IGHM, and AGT*), *TNF*^*+*^ macrophages (*TNF, IFNG, TGFB1, CCL3L3, CCL4L2, IL4, and IL13*), and *VSIG4*^*+*^ macrophages (*VSIG4, THBS1, CD163, MERTK, IFNGR1, and IFNAR1*). *S100A8*^*+*^ macrophages expressed high levels of *CX3CR1/CCR2* and were enriched in the circulation (Fig. 2B, C).

Notably, *C1Q*^*+*^ macrophages were highly expanded in the livers of sAH patients and expressed high levels of *MARCO* but low levels of *TIMD4*, suggesting that *C1Q*^*+*^ macrophages represent a transitional subtype that enters the liver from the circulation (Fig. 2B, C). Collectively, these scRNA-seq data reveal the heterogeneity of circulating and liver-infiltrating macrophages in sAH patients.

Our previous study also identified a subset of *C1Q*^*+*^ macrophages in a mouse model of acute liver injury, which functions to promote liver repair by removing necrotic tissue and dead cells [16]. To further explore the functions of *C1Q*^*+*^ macrophages in sAH, we analyzed genes related to phagocytic activity and cytokine secretion. *C1Q*^*+*^ macrophages expressed high levels of *MERTK, RAB7A, MSR1, IRF8, RAB20*, and *SCARB1* (Fig. 2D), genes involved in macrophage phagocytosis, indicating their strong phagocytic potential.

Interestingly, compared with other macrophage subsets, *C1Q*^*+*^ macrophages were the primary source of *IL10* (Fig. 2E) and expressed the highest levels of *IL6* and *IL1B* (Fig. 2E), suggesting that *C1Q*^*+*^ macrophages have both anti- and pro-inflammatory properties. In addition, *C1Q*^*+*^ macrophages expressed high levels of *GPNMB, TREM2, SPP1*, and *CD9* (Fig. 2C), resembling lipid-associated macrophages (LAMs) [14]. Furthermore, *C1Q*^*+*^ macrophages expressed high levels of *MSR1, SLC40A1, SRA1, HMOX1, PPARD*, and *ABCA1*, which are associated with iron and lipid metabolism (Fig. S2B, C). In summary, these findings demonstrate that *C1Q*^*+*^ macrophages play diverse roles in modulating inflammatory and phagocytic activities in ALD.

### Verification of macrophage signature gene expression via bulk RNA-seq and multiplex immunofluorescence staining

**Fig. 3:**
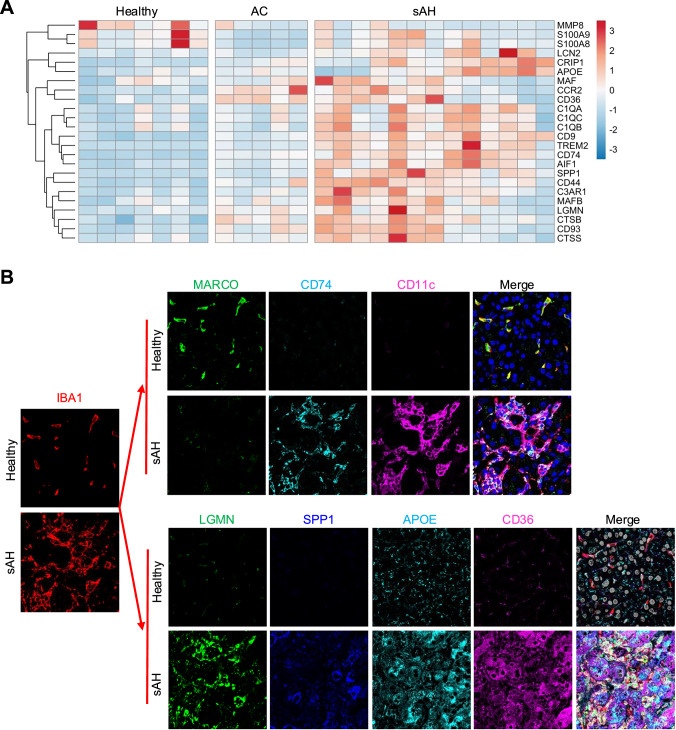
Verification of macrophage signature gene expression by bulk RNA-seq and multiplex immunofluorescence staining. **A** Bulk RNA-seq data from liver tissues of healthy controls and AC and sAH patients were analyzed. The heatmap displays macrophage signature genes in sAH patients compared with healthy controls and AC patients. **B** Liver tissues from healthy controls and sAH patients were subjected to multiplex immunofluorescence staining for IBA1, MARCO, CD74, and CD11c and LGMN, SPP1, APOE, and CD36

To validate the findings from scRNA-seq, we analyzed bulk RNA-seq data from healthy controls and AC and sAH patients. Most macrophage signature genes identified by scRNA-seq were upregulated in sAH patients (Fig. 3A). To further confirm these findings at the protein level, we performed multiplex immunofluorescence staining in sAH patient liver tissues (Fig. 3B). First, we examined the expression of macrophage surface markers and found that CD11C, a dendritic cell marker, was detected in some MoMFs and colocalized with CD74 (Fig. 3B, upper panel) in sAH patients.

Next, we analyzed proteins involved in specific macrophage functions (Fig. 3B, lower panel). LGMN, a lysosomal cysteine protease involved in protein degradation, was highly expressed in MoMFs. SPP1, a protein associated with C1Q^+^ macrophages, was also enriched in MoMFs. Additionally, MoMFs in sAH patients presented elevated expression of APOE and CD36, both of which are involved in lipid metabolism.

In summary, bulk RNA-seq analysis confirmed the upregulation of macrophage signature genes in sAH patients, while multiplex immunofluorescence staining further validated these findings at the protein level, revealing that MoMFs expressed CD11C, CD74, LGMN, SPP1, APOE, and CD36. These results suggest that MoMFs play diverse roles in sAH, including protein degradation and lipid metabolism, further highlighting their functional heterogeneity in liver pathology.

### Macrophage interactions with the accumulation of apoptotic neutrophils in sAH

**Fig. 4:**
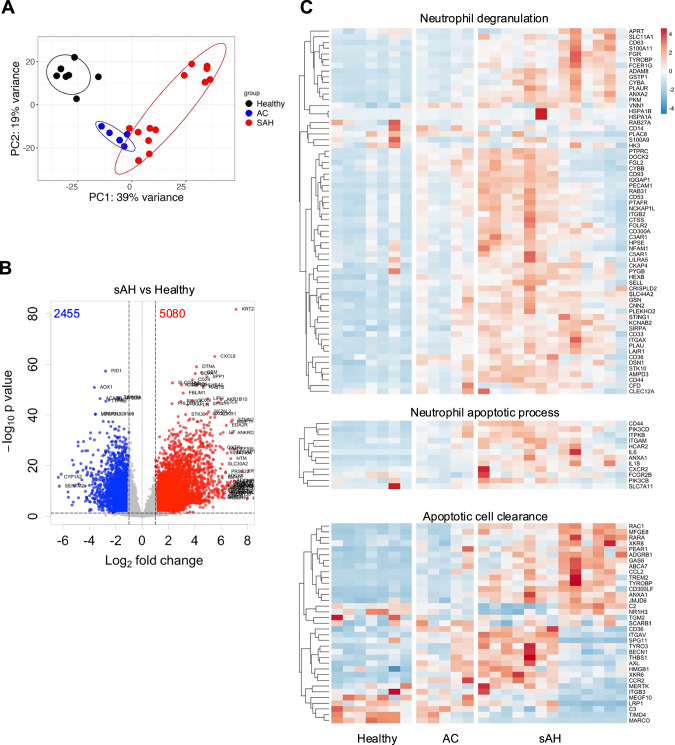
Bulk RNA-seq revealed differentially expressed genes in the livers of healthy controls and AC and sAH patients. **A** Bulk RNA-seq data of liver tissues from healthy controls and AC and sAH patients were analyzed. The PCA plot displays different gene expression patterns in healthy controls and AC and sAH patients. **B** Volcano plot showing differentially expressed genes in the livers of sAH patients and healthy controls. The x-axis indicates the log2-fold change (sAH patients vs. healthy controls), and the y-axis indicates the –log10 p value. The blue dots represent downregulated genes (log2[fold change] < −1, *p* < 0.05), and the red dots represent upregulated genes (log2[fold change] > 1, *p* < 0.05). **C** Heatmap showing the expression of neutrophil degranulation-related genes (upper panel), neutrophil apoptotic process-related genes (middle panel), and apoptotic cell clearance-related genes (lower panel) in the livers of healthy controls and AC and sAH patients

To gain deeper insights into the functions of liver macrophages in ALD, we analyzed our bulk RNA-seq data from healthy controls and AC and sAH patients[9]. Principal component analysis (PCA) revealed distinct clustering of gene expression patterns among these groups (Fig. 4A). Differential expression analysis revealed 5080 upregulated and 2455 downregulated genes in sAH patient livers compared with healthy control livers (Figs. 4B, S3). Further analysis revealed significant upregulation of genes associated with neutrophil degranulation, neutrophil apoptosis, and apoptotic cell clearance in sAH patients compared with both healthy controls and AC patients (Fig. 4C). These findings suggest an accumulation of apoptotic neutrophils in ALD, prompting us to investigate the interactions between neutrophils and macrophages in sAH.

**Fig. 5:**
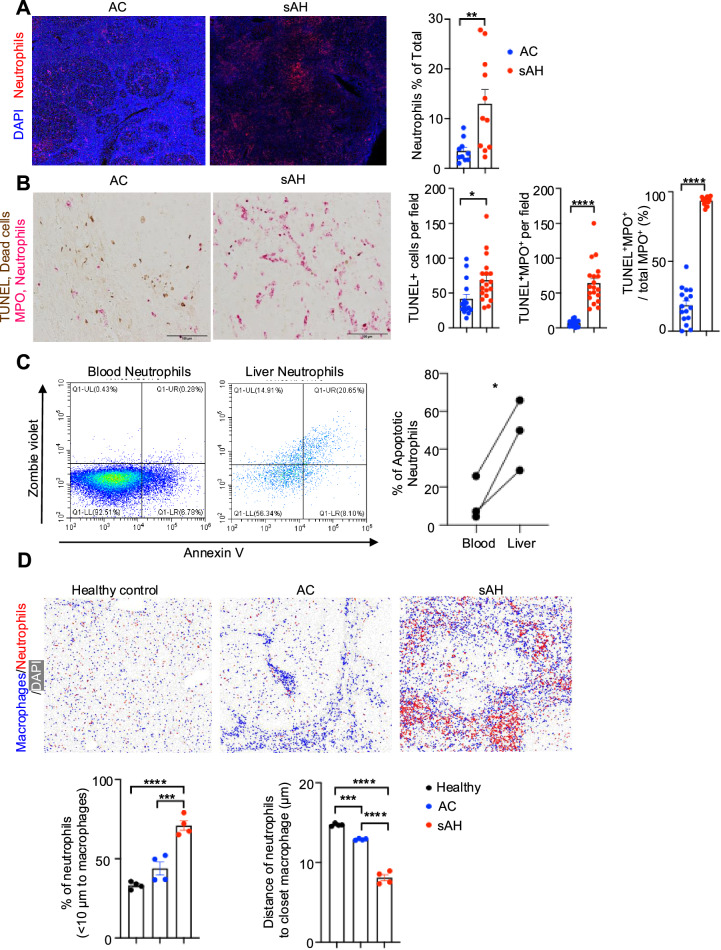
Accumulation of apoptotic neutrophils in sAH. **A** Liver tissues from sAH and AC patients were subjected to immunofluorescence staining of neutrophils (red) and DAPI (blue). Representative images (left) and the percentage of neutrophils among total liver cells (right) are shown. **B** Liver tissues from sAH and AC patients were subjected to TUNEL (brown) and MPO (pink) double staining. Representative images are shown. Scale bars, 100 µm. The quantification of TUNEL^+^ cells and TUNEL^+^MPO^+^ cells per field, and the percentage of TUNEL^+^MPO^+^ cells among the total number of MPO^+^ cells are shown. **C** Neutrophils were isolated from the fresh peripheral blood and liver of sAH patients and were immediately analyzed by staining with Zombie Violet and Annexin V via flow cytometry. The percentage of apoptotic neutrophils is shown on the right. **D** Spatial distribution of IBA1^+^ cells and MPO^+^ cells in liver tissues from healthy controls and AC and sAH patients (upper). The percentage of neutrophils located within 10 µm of a macrophage (lower left) and the average distance from the neutrophil to the closest macrophage (lower right) are shown. The values are presented as the means ± SEMs. *P < 0.05, **P < 0.01, ***P < 0.001, as determined by 2-tailed Student’s *t* test for comparing 2 groups (**A**–**C**) or 1-way ANOVA followed by Tukey’s post hoc test for multiple groups (**D**)

Our recent studies revealed massive neutrophil accumulation in the livers of sAH patients, where these neutrophils produced IL-8 to recruit additional neutrophils, thereby sustaining inflammation [9]. Immunofluorescence staining revealed that neutrophils accounted for more than 10% of the total liver cells in sAH patients, whereas they constituted less than 5% of the total liver cells in AC patients (Fig. 5A). To further investigate the fate of accumulated neutrophils, we performed TUNEL and MPO double staining on liver samples from AC and sAH patients. As shown in Fig. 5B, sAH patients presented a significantly greater number of TUNEL^+^ cells than AC patients did, with most of these apoptotic cells identified as neutrophils. Further analysis revealed that approximately 90% of MPO^+^ neutrophils were TUNEL^+^ in sAH patients, whereas in AC patients, TUNEL^+^ neutrophils accounted for only ~20% of the total neutrophil population.

Additionally, we assessed apoptotic markers in neutrophils from both the peripheral blood and liver of sAH patients. The proportion of apoptotic neutrophils in the liver was significantly greater than that in circulating WBCs (Fig. 5C). These data strongly suggest that massive neutrophil apoptosis occurs in the livers of sAH patients, contributing to disease pathology.

To further investigate macrophage‒neutrophil interactions, we analyzed the spatial distributions of IBA1^+^ macrophages and MPO^+^ neutrophils in liver tissues from healthy controls and AC and sAH patients (Fig. 5D). Our analysis revealed that in sAH patients, neutrophils were in closest proximity to macrophages, with approximately 60–70% of neutrophils located within 10 µm of a macrophage. In contrast, this percentage was less than 50% in healthy controls and AC patients. Additionally, the average distance between neutrophils and the nearest macrophage was less than 8 µm in sAH patients, whereas it was greater than 15 µm in healthy controls and 12 µm in AC patients. This close spatial association suggests strong macrophage‒neutrophil interactions in sAH, further supporting their role in disease pathology.

### *C1q* KO mice exhibit alcohol-induced liver injury similar to that of WT mice

**Fig. 6:**
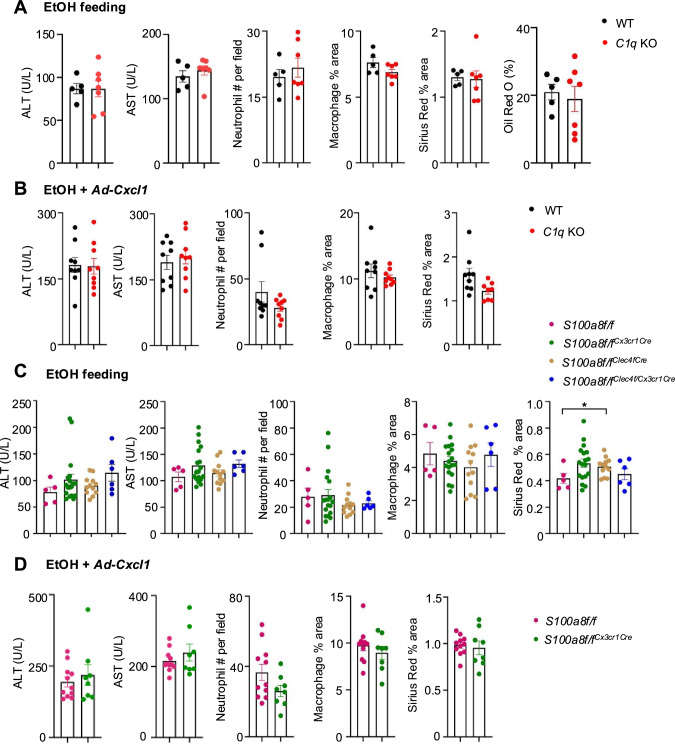
Comparison of liver injury and neutrophil infiltration in WT, *C1q* KO, and macrophage-specific S100a8 KO mice in the EtOH-fed model. **A** WT mice and *C1q* KO mice were subjected to the chronic-plus-binge ethanol model. **B** WT mice and *C1q* KO mice were subjected to chronic-plus-binge ethanol and the Ad-*Cxcl1* model. **C** WT, macrophage-specific *S100a8* KO mice (*S100a8f/f*^*Cx3cr1Cre*^), Kupffer cell-specific *S100a8* KO (*S100a8f/f*^*Clec4fCre*^), and mice with both Kupffer cell- and macrophage-specific *S100a8* knockout (*S100a8f/f*^*Clec4fCreCx3cr1Cre*^) were subjected to the chronic-plus-single binge model. **D** WT mice and macrophage-specific *S100a8* KO mice (*S100a8f/f*^*Cx3cr1Cre*^) were subjected to chronic-plus-binge ethanol and the Ad-*Cxcl1* model. Serum ALT and AST levels were measured. Liver tissues were subjected to HE staining, IHC staining of neutrophils and macrophages, Sirius Red staining and Oil Red O staining. The results of the quantification of neutrophils, macrophages, and Sirius Red and Oil Red O staining are shown. The values are presented as the means ± SEMs. *P < 0.05, **P < 0.01, ***P < 0.001, as determined by 2-tailed Student’s *t* test for comparisons between 2 groups

C1q^+^ macrophages have been implicated in promoting necrotic lesion resolution following acute liver injury [16]. To assess their role in ALD, we utilized a 10-day chronic-plus-binge ethanol-feeding model in both WT and *C1q* KO mice. As shown in Fig. 6A, both groups presented comparable levels of liver injury and hepatic neutrophil infiltration, as indicated by similar serum ALT and AST levels, macrophage and neutrophil infiltration, fibrosis and steatosis. The lack of exacerbated liver damage in *C1q* KO mice may be attributed to the mild liver injury and low levels of apoptosis observed in this model.

We previously demonstrated that CXCL1, CXCL6, and IL-8 are upregulated in sAH patients [9], whereas compared with humans, mice exhibit significantly lower neutrophil counts and lack key neutrophil chemokines such as IL-8, resulting in lower CXCL1 levels [17]. Consistently, liver-specific *Cxcl1* knockout in mice did not significantly impact alcohol-induced liver injury (Fig. S4A, B), likely due to inherently low endogenous CXCL1 levels and only mild elevation after alcohol exposure.

To better replicate AH patients with elevated neutrophil chemokines, we utilized an ethanol-feeding model with *Cxcl1* overexpression [17, 18]. As shown in Fig. 6B, the combination of ethanol feeding and *Cxcl1* overexpression led to a significant increase in the serum ALT and AST levels in both the WT and *C1q* KO mice compared with those in their respective controls. Both WT and *C1q* KO mice in the EtOH + *Cxcl1* group presented similarly elevated ALT and AST levels.

Given that C1q is produced primarily by macrophages [19] and plays a crucial role in clearing apoptotic cells [20], along with insights from scRNA-seq data, our findings suggest that C1q^+^ macrophages may have dual roles in alcohol-induced liver injury, potentially influencing both tissue damage and repair processes.

### Macrophage-specific *S100a8* knockout mice exhibit alcohol-induced liver injury similar to that of WT mice

S100A8 is produced predominantly by neutrophils and macrophages and has been extensively studied in various inflammatory diseases, including infection-induced inflammation, metabolic inflammation, immune system dysfunction, and degenerative diseases [21]. Our scRNA-seq data identified a subset of *S100a8*^*+*^ macrophages, but the role of macrophage-derived S100a8 in ALD remains unclear.

To investigate this, we generated macrophage-specific *S100a8* KO mice (*S100a8*f/f^*Cx3cr1*Cre^), Kupffer cell-specific *S100a8* KO mice (*S100a8*f/f^*Clec4f*Cre^), and mice with both Kupffer cell- and macrophage-specific *S100a8* knockout (*S100a8*f/f^*Clec4f*Cre*Cx3cr1*Cre^). These knockout mice, along with their WT littermate control *S100a8*f/f mice, were subjected to a 10-day chronic-plus-binge ethanol-feeding regimen. As shown in Fig. 6C, all groups exhibited comparable levels of liver injury and hepatic neutrophil infiltration, as indicated by similar serum ALT and AST levels, macrophage and neutrophil infiltration, and fibrosis. Additionally, WT and *S100a8*f/f^*Cx3cr1*Cre^ mice presented similar steatosis patterns (Figure. S5A). These findings suggest that the absence of Kupffer cell- and macrophage-derived *S100a8* does not influence ethanol-induced liver damage.

Alcohol-associated liver disease in mice is generally milder than the severely inflamed liver seen in human sAH, and this may not induce the expression of *S100A8* in the recruited macrophages. To explore this further, we subjected both WT and *S100a8*f/f^*Cx3cr1*Cre^ mice to ethanol-feeding model with *Cxcl1* overexpression. As shown in Fig. 6D, the combination of ethanol feeding and *Cxcl1* overexpression induced higher levels of serum ALT and AST than ethanol-fed mice did; however, similar liver damage was observed in both WT and *S100a8*f/f^*Cx3cr1*Cre^ mice, as indicated by serum ALT and AST levels, macrophage and neutrophil infiltration, and fibrosis. We also tested the ethanol plus LPS model and found that liver injury and inflammation were comparable in WT and *S100a8*f/f^*Cx3cr1*Cre^ mice (Fig. S5B).

These findings suggest that macrophage-specific *S100a8* deletion does not significantly alter alcohol-induced liver injury, likely due to the relatively mild inflammatory response in murine models compared with the more severe immune activation observed in human sAH, where S100A8⁺ macrophages are prominently enriched. Another possible explanation is that S100a9 may form homodimers that partially compensate for the loss of S100a8. Given that S100a8/S100a9 heterodimers and S100a9 homodimers may possess distinct and/or redundant biological functions, such compensatory mechanisms could obscure the impact of S100a8 deficiency under the current experimental conditions.

### *Apoe* KO mice are more susceptible to alcohol-induced liver injury

**Fig. 7:**
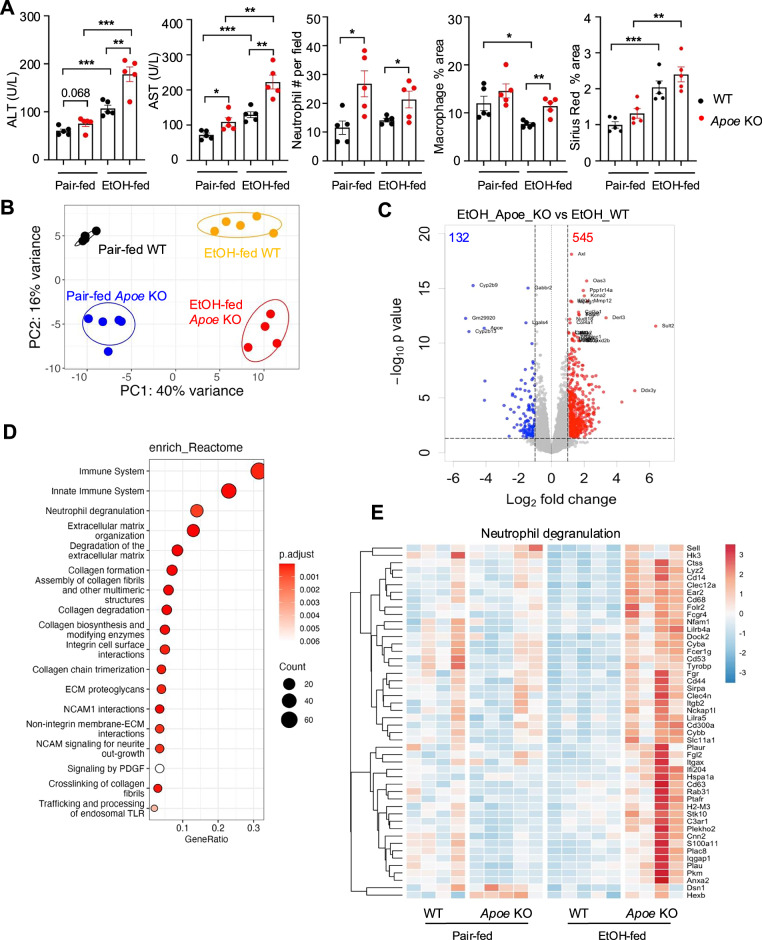
Comparison of liver injury and neutrophil infiltration in WT and *Apoe* KO mice in the EtOH-feeding model. **A** WT mice and *Apoe* KO mice were subjected to the chronic-plus-single binge model. Serum ALT and AST levels were measured. Liver tissues were subjected to HE staining, IHC staining of neutrophils and macrophages, and Sirius Red staining. The quantification of neutrophils, macrophages, and Sirius Red is shown. **B** Liver tissues from WT and *Apoe* KO mice were subjected to bulk RNA-seq analysis. The PCA plot shows different gene expression patterns between pair-fed and EtOH-fed *Apoe* KO and WT mice. **C** Volcano plot displaying differentially expressed genes in the livers of EtOH-fed *Apoe* KO and WT mice. The x-axis represents the log2-fold change *(Apoe* KO vs. WT), and the y-axis represents the –log10 p value. The blue dots represent downregulated genes (log2[fold change] < −1, *p* < 0.05), and the red dots represent upregulated genes (log2[fold change] > 1, *p* < 0.05). **D** Reactome pathway analysis of differentially expressed genes in the livers of EtOH-fed *Apoe* KO and WT mice. **E** Heatmap showing the expression of neutrophil degranulation-related genes in the livers of *Apoe* KO and WT mice. The values are presented as the means ± SEMs (**A**, **B**). *P < 0.05, **P < 0.01, ***P < 0.001, as determined by a 2-tailed Student’s *t* test for comparing two groups

Our previous data revealed that *C1q*^+^ macrophages from a ConA-induced liver injury model express *Apoe* [16], but the role of *Apoe* in liver repair remains unexplored. Interestingly, both *C1Q*^*+*^ macrophages and *APOE*^*+*^ macrophages in sAH patients presented high *APOE* expression (Fig. 2B, C). To investigate the role of *Apoe* in ALD, we subjected WT and *Apoe* knockout (*Apoe* KO) mice to a 10-day chronic-plus-binge ethanol feeding model. As shown in Fig. 7A and Fig. S6A, *Apoe* KO mice presented increased susceptibility to alcohol-induced liver injury, inflammation and steatosis, as evidenced by significantly increased serum ALT and AST levels, increased hepatic neutrophil infiltration, exacerbated fibrosis and Oil Red O staining.

To further explore the molecular mechanisms underlying the enhanced liver damage in *Apoe* KO mice, we performed RNA sequencing analysis on liver tissues from pair-fed and ethanol-fed WT and *Apoe* KO mice. As illustrated in Fig. 7B, PCA revealed distinct clustering of gene expression patterns between pair-fed and ethanol-fed mice, as well as between WT and *Apoe* KO mice, suggesting a significant alteration in the transcriptomic profile of ethanol-fed *Apoe* KO mice. Differential expression analysis revealed 545 genes whose expression was significantly upregulated and 132 genes whose expression was downregulated in the livers of ethanol-fed *Apoe* KO mice compared with those of ethanol-fed WT mice (Figs. 7C, S6B).

Notably, Reactome pathway analysis highlighted several key pathways that were altered in ethanol-fed *Apoe* KO mice, including neutrophil degranulation, extracellular matrix organization, and collagen formation (Fig. 7D). Among these genes, neutrophil degranulation-related genes were significantly upregulated in ethanol-fed *Apoe* KO mice (Fig. 7E). Given the critical role of macrophage-derived *Apoe* in the resolution of lung fibrosis [22] and the increase in liver fibrosis observed in ethanol-fed *Apoe* KO mice (Fig. 7A), *Apoe*^+^ macrophages may contribute to the resolution of liver fibrosis. Further studies using macrophage-specific *Apoe*-KO mice are necessary to validate this hypothesis.

## Discussion

**Fig. 8:**
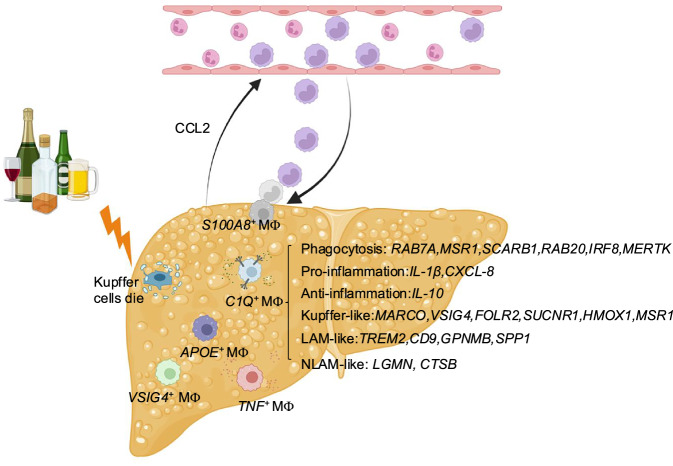
Schematic of the diverse functions of liver macrophages in severe alcohol-associated hepatitis. Heavy alcohol consumption damages hepatocytes, leading to the release of CCL2, which attracts numerous infiltrating monocyte-derived macrophages (MoMFs) to the injured liver to replace Kupffer cells. MoMFs include *C1Q*^*+*^ macrophages, *APOE*^*+*^ macrophages, *TNF*^*+*^ macrophages, *VSIG4*^*+*^ macrophages, and *S100A8*^*+*^ macrophages. *C1Q*^*+*^ macrophages are unique in sAH and express various groups of genes that likely play various roles in controlling AH disease progression *(Created with Biorender.com)*

Although macrophage subtypes have been extensively studied via scRNA-seq analysis [14, 23], liver macrophage populations in AC and sAH were not examined until our current study, in which we provide a comprehensive characterization of liver macrophages in AC and sAH via scRNA-seq, bulk RNA-seq, and multiplex immunofluorescence analyses. We identified several distinct macrophage populations, including *C1Q*^+^, *S100A8*^+^, *APOE*^+^, *TNF*^+^, and *VSIG4*^+^ macrophages (Fig. 8). Notably, both AC and sAH were associated with an ~90% reduction in Kupffer cells (KCs), which are largely replaced by monocyte-derived macrophages (MoMFs). MoMFs in sAH presented high expression of a diverse range of signature genes, suggesting their active involvement in disease pathogenesis. Our findings indicate that *C1Q*⁺ macrophages may play complex roles in ALD. Additionally, we observed a significant accumulation of apoptotic neutrophils in sAH livers, with many in close proximity to macrophages, highlighting a potential interaction that may influence disease progression.

Previously, we demonstrated that sAH livers were heavily infiltrated by IBA1⁺ macrophages [8]. More recently, we established that IBA1 and CLEC4F double staining provides a more reliable method than F4/80 staining alone for distinguishing KCs from other macrophages in both healthy and diseased mouse livers [24]. In this study, we further employed IBA1 and MARCO double staining to visualize KCs and MoMFs in liver sections from healthy controls and AC and sAH patients, providing compelling evidence that KCs are largely replaced by MoMFs in sAH. KC replacement by MoMFs may hamper the recovery of liver function and increase bacterial infections, leading to immunosuppression [25].

Through the integration of internal and external liver RNA-seq data, we report for the first time that classical KC markers (*MARCO, CD5L*, and *TIMD4*) are significantly downregulated in AC and sAH, correlating negatively with MELD scores. In contrast, MoMF markers (*TREM2, GPNMB*, and *SPP1*) are upregulated and positively correlated with MELD scores, further illustrating the dynamic shifts in macrophage populations during disease progression. Interestingly, these macrophage markers did not effectively differentiate patients with other liver disease etiologies from healthy controls. The anticipated upregulation of *TREM2*, *GPNMB*, and *SPP1* in our RNA-seq datasets of metabolic dysfunction-associated steatotic liver disease (MASLD) was less pronounced than expected, despite previous studies consistently identifying lipid-associated macrophages (LAMs) as highly expressing *TREM2*, *CD9*, *GPNMB*, and *SPP1* [26–30]. One possible explanation is that the proportion of MoMFs relative to total liver macrophages in MASLD or metabolic dysfunction-associated steatohepatitis (MASH) is not as drastically altered as that in sAH. This finding warrants further investigation to clarify the extent of MoMF expansion across different liver disease etiologies.

To the best of our knowledge, this is the first comprehensive study to characterize liver macrophages in AC and sAH via an integrated approach combining scRNA-seq, bulk RNA-seq, and multiplex immunofluorescence staining. Our findings offer valuable insights into macrophage heterogeneity and their roles in ALD, paving the way for future investigations into targeted therapeutic strategies.

### *C1Q*^+^ macrophages in sAH

C1Q, which is produced primarily by macrophages, is a soluble protein in the circulation and functions as an opsonin, enhancing apoptotic cell engulfment [31]. It can also function as a pattern recognition receptor (PRR) on macrophage membranes, bridging apoptotic cells to phagocytes [31]. Historically, research has focused on C1Q molecules rather than C1Q-producing macrophages [32–34]. With advancements in scRNA-seq, *C1Q*⁺ macrophages have been identified in various diseases, including cancers [34–37], allergic lung inflammation [38], acute myeloid leukemia [39], bullous pemphigoid [40], and Behçet’s disease [41]. Their roles vary depending on the tissue and disease context. In our study, *C1Q*⁺ macrophages were uniquely predominant in sAH patients. Given the accumulation of apoptotic neutrophils in sAH livers—where neutrophils are closest to macrophages and predominantly within 10 µm—*C1Q*⁺ macrophages likely play a role in clearing apoptotic neutrophils. In support of this, we previously demonstrated that *C1q*⁺ macrophages promote necrotic liver lesion resolution by removing dead cells in a model of ConA-induced acute liver injury [16, 42]. Many genes involved in necrotic cell clearance in the ConA model were also expressed in *C1Q*⁺ macrophages from sAH patients, as demonstrated in the current study.

Additionally, *C1Q*⁺ macrophages express Kupffer cell markers (*MARCO, FOLR2, SUCNR1, GFRA2, TMEM26, HMOX1, and TIMD4*), proinflammatory genes (*IL-1B* and *CXCL8/IL-8*), and anti-inflammatory genes (*IL-10*). They also highly express genes involved in iron and lipid metabolism, resembling resident Kupffer cells in other liver diseases [27–30, 43]. Moreover, *C1Q*^+^ macrophages in sAH express *GPNMB, TREM2, SPP1*, and *CD9*, resembling LAMs, which are reported in liver cirrhosis [44] and hepatocellular carcinoma (HCC) [45]. However, it remains uncertain whether LAMs represent an endpoint of macrophage differentiation or if they can transform into monocyte-derived Kupffer cells (moKCs) or vice versa [14]. Overall, we conclude that *C1Q*^+^ macrophages likely play various roles in sAH development and progression.

To further understand the role of *C1Q* in macrophages in ALD, we performed chronic-plus-binge ethanol feeding and chronic-plus-binge ethanol feeding plus the overexpression of *Cxcl1* in WT and *C1q*KO mice. Alcohol-induced liver injury and neutrophil infiltration were increased in both WT and *C1q*KO mice with *Cxcl1* overexpression, but no statistically significant differences were observed between these two mouse lines. The lack of a phenotype in *C1q* KO mice after alcohol exposure may be due to mild liver injury and inflammation in the ALD models we used.

### *S100A8*^*+*^ macrophages

The S100a8 and S100a9 proteins function primarily as heterodimers known as calprotectin [21]. While both can be expressed independently, their inflammatory potency is significantly enhanced when they are dimerized. In *S100a8*-deficient mice, residual S100a9 homodimer activity may partially compensate, potentially masking a stronger phenotype. Our findings should therefore be interpreted in the context of this redundancy, and future studies using double knockout models or calprotectin-specific inhibitors would provide more definitive insights.

### Role of *APOE*^+^ macrophages

Both *C1Q*^+^ and *APOE*^+^ macrophages express *ApoE*, as shown in this study and in our previous acute liver injury model in mice. APOE has anti-inflammatory roles in modulating macrophage behavior [46, 47] and regulating neutrophils, as observed in atherosclerosis [48]. We demonstrated that *Apoe* KO mice are more susceptible to alcohol-induced injury and fibrosis, which indicates the anti-inflammatory role of *Apoe* in ALD. While we demonstrated that *Apoe* KO mice are more susceptible to alcohol-induced injury and fibrosis, we did not generate macrophage-specific *Apoe* KO mice to directly assess the role of macrophage-derived *Apoe*. Given that a previous study revealed a critical role for macrophage *Apoe* in the resolution of lung fibrosis [22], we hypothesize that *APOE*^+^ macrophages may similarly promote the resolution of liver fibrosis in alcohol-induced liver injury.

### Therapeutic implications

Macrophages have been actively explored as therapeutic targets for liver diseases [49, 50]. Thus, we asked whether a specific subset(s) of macrophages can be used as therapeutic targets for AH. AH-specific IL-8⁺ neutrophils accumulate in AHs but not in ACs [9], making them potential therapeutic targets [11]. In contrast, the differences in macrophage populations between AHs and ACs are less distinct than those in neutrophils between AHs and ACs. Interestingly, AH patients uniquely exhibit *C1Q*^+^ macrophages, which may play both detrimental (promoting inflammation) and beneficial (removing dead cells) roles, making them unsuitable as direct therapeutic targets. However, given the widespread infiltration of macrophages in both AHs and ACs, reducing macrophage trafficking into the liver may provide therapeutic benefits. Blockade of macrophage infiltration and activation has been investigated in preclinical models and patients with MASH [49], and some of these approaches can be used to test the inhibition of macrophages in AH and AC patients in the future.

## Materials and methods

### Mice

C57BL/6 J wild-type (WT) mice *and C1qa* KO (JAX: 031675), *Apoe* KO (JAX: 002052), *Cx3cr1*-Cre (JAX: 025524), *Clec4f-*Cre (JAX: 033296) and *Alb*-Cre (JAX: 003574) mice were purchased from The Jackson Laboratory (Bar Harbor, Maine, USA). *Cxcl1*f/f mice were generated by introducing LoxP sites flanking exon 2 of the *Cxcl1* gene (Applied StemCell, Inc.). *Cxcl1*f/f^*AlbCre*^ mice were generated by crossing *Cxcl1*f/f mice with *Alb*-Cre mice via several steps. *S100a8f/f* mice were previously described [51]. *S100a8*f/f^*Clec4f*Cre^ and *S100a8*f/f^*Cx3cr1*Cre^ were generated by crossing *S100a8f/f* mice with *Clec4f*-Cre mice and *Cx3cr1*-Cre mice via several steps, respectively. *S100a8*f/f^*Clec4f*Cre*Cx3cr1*Cre^ mice were generated by crossing *S100a8*f/f^*Clec4f*Cre^ and *S100a8*f/f^*Cx3cr1*Cre^ mice via several steps.

The mice in the chronic-plus-binge ethanol-feeding model were fed as described in our previous study [52]. Briefly, 10–12-week-old female or male mice were acclimated by ad libitum feeding with a Lieber–DeCarli liquid diet (F1259SP, Bio-Serv) for five days. Following acclimatization, the ethanol-fed groups received a Lieber–DeCarli ethanol diet (F1258SP, Bio-Serv) containing 5% (v/v) ethanol for 10 days. On day 11, a single dose of ethanol (5 g/kg body weight) was administered via oral gavage in the early morning, and the mice were euthanized nine hours later. Pair-fed control mice were maintained on an isocaloric control diet for 10 days and, on the day of euthanasia, received an oral gavage of isocaloric dextrin-maltose (9 g/kg body weight).

We also employed a chronic-plus-binge ethanol-feeding model combined with *Cxcl1* overexpression to increase neutrophil infiltration [17, 18]. After a five-day acclimatization period with the control diet, the mice received a single tail vein injection of adenovirus expressing *Cxcl1* (Ad-Cxcl1) at the beginning of ethanol feeding. The mice were then fed an ethanol diet for 14 days and administered a single ethanol gavage on day 15. Nine hours post-gavage, the mice were euthanized for analysis.

All animal experiments were approved by the National Institute on Alcohol Abuse and Alcoholism’s (NIAAA) Animal Care and Use Committee under protocol LLD-BG-01.

### Single-cell RNA sequencing (scRNA-seq) and bulk RNA sequencing of human samples

Liver and blood samples were obtained from patients with sAH and AC from the Department of Surgery at Johns Hopkins Hospital, supported by the NIAAA (R24AA025017, Clinical Resources for AH Investigators). The scRNA-seq data on liver and peripheral white blood cells from healthy controls and AC and sAH patients were previously deposited in the Gene Expression Omnibus (GEO) with accession number GSE255772 [9]. Bulk RNA sequencing data of liver tissues from healthy controls and AC and sAH patients were also deposited in the GEO database as GSE143318 [9]. Additionally, we analyzed bulk RNA-seq data (phs001807.v1.p1) from another cohort [15].

### Bulk RNA sequencing of mouse samples

Total RNA was extracted from the livers of pair-fed and ethanol-fed WT and *Apoe* KO mice in the chronic-plus-binge model via TRIzol (Thermo Fisher Scientific). Sequence reads were analyzed on the NIH HPC Biowulf cluster (http://hpc.nih.gov) via updated versions of Trimmomatic, HISAT2, and FeatureCounts. Differential expression analysis was conducted via the DESeq2 package in R. Pathway analysis and heatmap generation were performed via the R packages clusterProfiler and pheatmap. The RNA-seq data have been deposited in the GEO under accession GSE292193.

### Immunohistochemical staining

Liver tissues were collected and fixed in 10% buffered formalin, followed by paraffin embedding. Sections (2.5 μm) were cut for Sirius Red staining (Millipore Sigma) or immunohistochemical (IHC) staining. For IHC, after heat-induced epitope retrieval in buffer (1 mM EDTA, 10 mM Tris-HCl pH 9, and 10% glycerol), the paraffin-embedded sections were stained with primary antibodies and visualized via the DAB Peroxidase Substrate Kit (SK-4105, Vector Laboratories). Images were acquired via a whole-slide scanner (Aperio VERSA, Leica Biosystems). Positive cells and positive areas in five randomly selected high-power fields (1250 μm × 1250 μm/field) were analyzed via QuPath and ImageJ.

### Multiplex immunofluorescence staining

Human liver sections (2.5 μm) were prepared for multiplex immunofluorescence staining with more than three markers, as previously described [53]. The acquired images were processed and analyzed via ImageJ [54] and the FIJI plugin HyperStackReg V5.682, along with Ilastik (version 1.3.3post3) [55] and CellProfiler (version 4.2.6) [56] for cell counting and quantification, as previously described [13]. The extracted cell location and mean fluorescence intensity data were plotted and visualized via GraphPad Prism (version 10.0).

### Apoptotic neutrophil detection

TUNEL and MPO double-staining of the liver was performed on human liver sections via the ApopTag Peroxidase In Situ Apoptosis Detection Kit (S7100, Sigma‒Aldrich) following the manufacturer’s instructions. TUNEL staining was combined with immunohistochemical staining for MPO.

For flow cytometry analysis, single-cell suspensions of liver mononuclear cells (MNCs) or white blood cells (WBCs) were washed in PBS containing 1% bovine serum albumin. Apoptotic neutrophils were assessed via Zombie Violet (BioLegend) and Annexin V (Thermo Fisher Scientific) according to the manufacturer’s instructions.

**Antibody**

**Table:** 

Antibody	Vendor	Cat. No.	Dilution
IBA1	Wako	019-19741	1:1000
MARCO	Invitrogen	PA5-64134	1:200
CD74	OriGene	AM32811PU-T	1:200
CD11c	CST	45581	1:200
LGMN	CST	93627S	1:200
SPP1	CST	88742	1:200
APOE	CST	13366	1:200
CD36	Invitrogen	MA1-46000	1:200
MPO	DSHB	CPTC-MPO-1	1:200
S100A9	CST	73425	1:200
F4/80	CST	70076	1:200
TIM4	CST	75484	1:200
CD68	Biocare Medical	KP1	1:200

### Statistical analysis

The data were analyzed via GraphPad Prism 10 (GraphPad Software) and are presented as the means ± standard errors of the means (SEMs), unless otherwise specified. Statistical comparisons were conducted via appropriate tests, including Student’s *t* test for two-group comparisons and one-way ANOVA followed by Tukey’s post hoc test for multiple-group comparisons. A *p* < 0.05 was considered statistically significant.

## Supplementary information

Fig. S1-6 and Table S1
