# Supplementary material for: Infiltrating macrophages replace Kupffer cells and play diverse roles in severe alcohol-associated hepatitis
Source: Cell Mol Immunol. 2025 Sep 17;22(10):1262–75. doi: 10.1038/s41423-025-01343-1 (PMC12480828; doi:10.1038/s41423-025-01343-1)
Supplement: Supplementary file 1 — Fig. S1-6 and Table S1 [file 41423_2025_1343_MOESM1_ESM.pdf]

Table S1. Clinical and Demographic Characteristics of Patients for scRNA-seq Included in the Study

ID	Age	Sex	Race/Ethnicity	Meld-Na on Tx day	Drinking History (Sobriety <3m, <6m, >6m before Tx)	Steroid Tx before surgery at Hopkins
AH 100	49	M	White/Not Hispanic	32	<3m	10/25/2022
AH 101	50	M	White/Not Hispanic	41	<3m	n/a
AH 102	50	M	Black/Not Hispanic	31	<3m	n/a
AH 103	59	M	Black/Not Hispanic	38	<3m	n/a
AH 104	39	M	White/Not Hispanic	45	<3m	n/a
AC 27	59	M	White/Not Hispanic	24	>6m	5.6
AC 28	64	F	White/Not Hispanic	16	>6m	2.3
AC 29	41	M	White/Not Hispanic	21	>6m	3.6

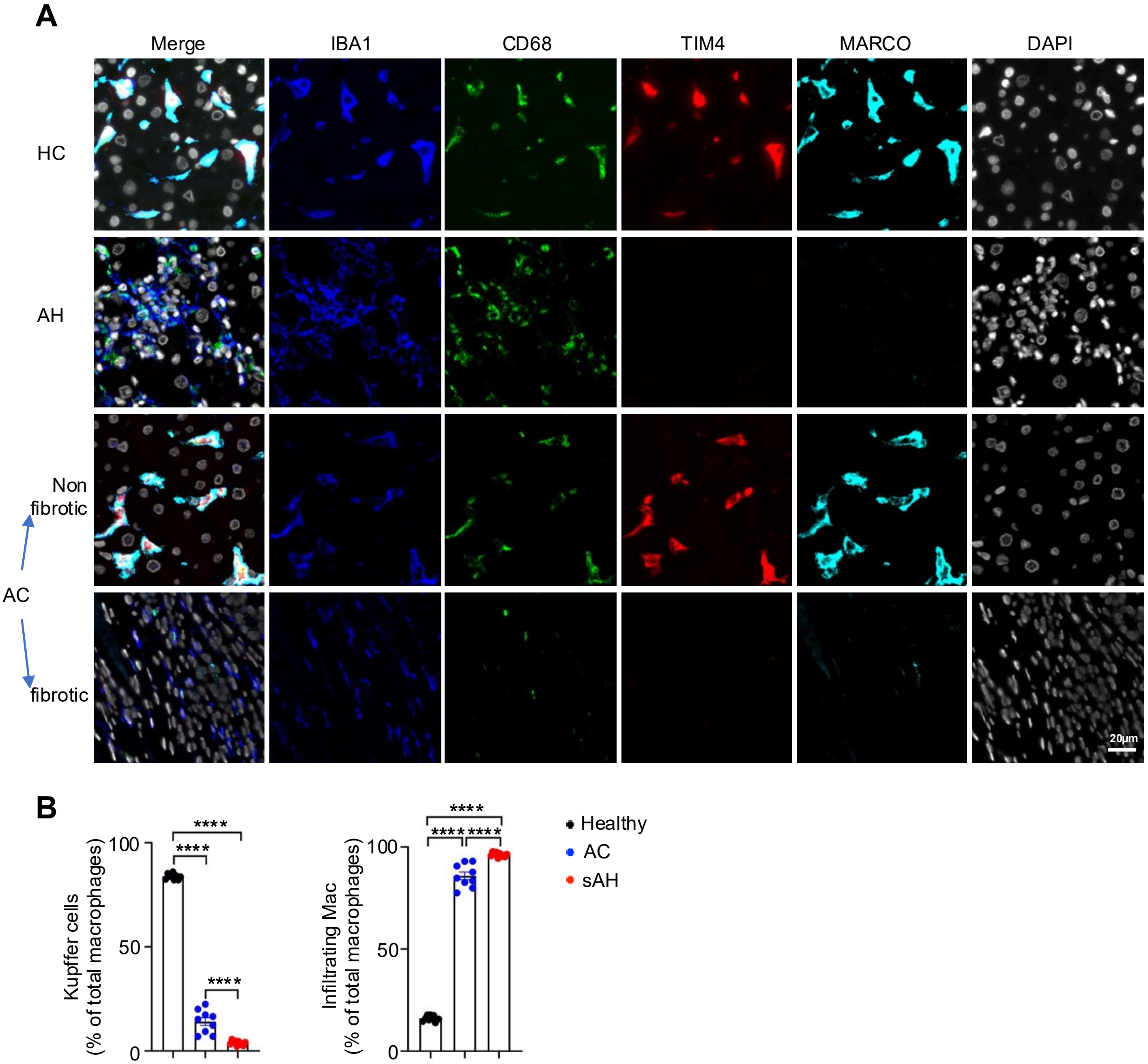

Fig. S1. (A) Liver tissues from healthy controls, AC and sAH patients were analyzed using multiplex immunofluorescence staining for IBA1/CD68/TIM4/MARCO. Representative immunofluorescence images are shown. Scale bars, 20μm. (B) The percentages of Kupffer cells (left) and infiltrating macrophages (right) among total IBA1$^+$ cells related to Fig 1A and 1B.

C

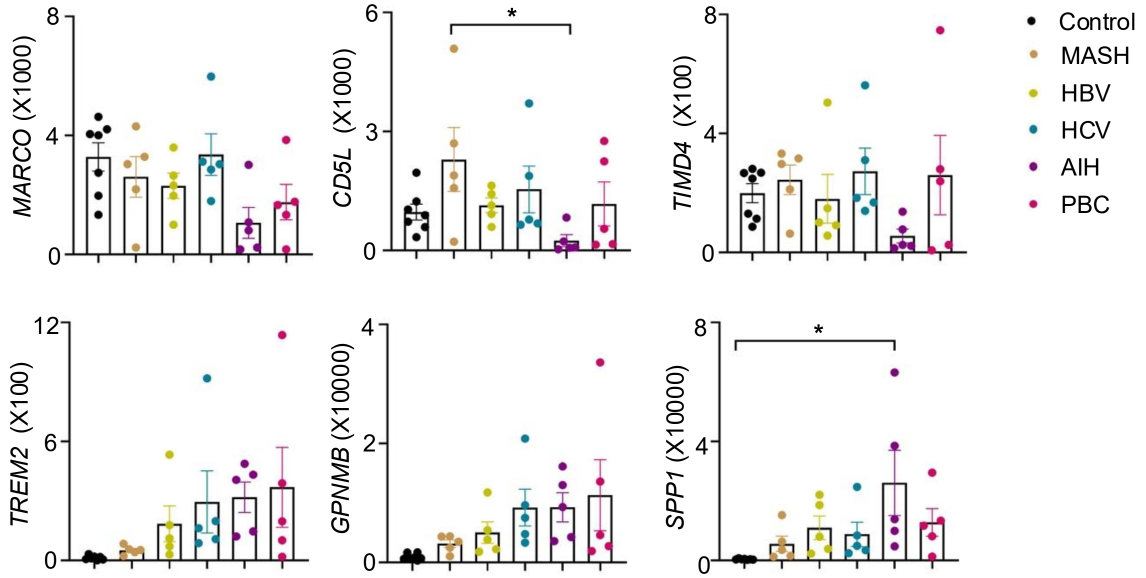

D

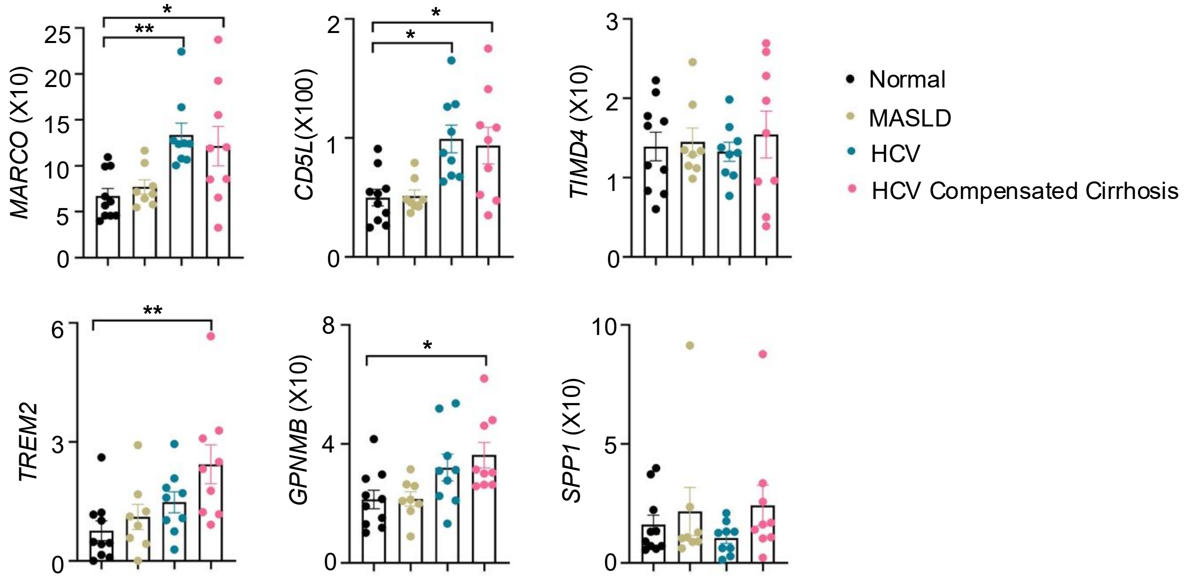

Fig. S1. (C) Hepatic expression levels of Kupffer cell markers (*MARCO, CD5L, and TIMD4*) and macrophage markers (*TREM2, GPNMB, and SPP1*) were compared between healthy controls (n=7), MASH (n=5), HBV (n=5), HCV (n=5), AIH (n=5), and PBC (n=5) based on RNA-seq data. Gene expression levels are presented as relative read counts. (D) Hepatic expression of Kupffer cell and infiltrating macrophage markers was analyzed using RNA-seq data from healthy controls (n=10), MASLD (n=8), HCV (n=9), and HCV_Compensated Cirrhosis (n=9). Gene expression levels are presented as transcripts per million. Values are represented as mean ± SEM. *$P < 0.05$, **$P < 0.01$, ***$P < 0.001$, as determined by 1-way ANOVA followed by Tukey's post hoc test for multiple groups.

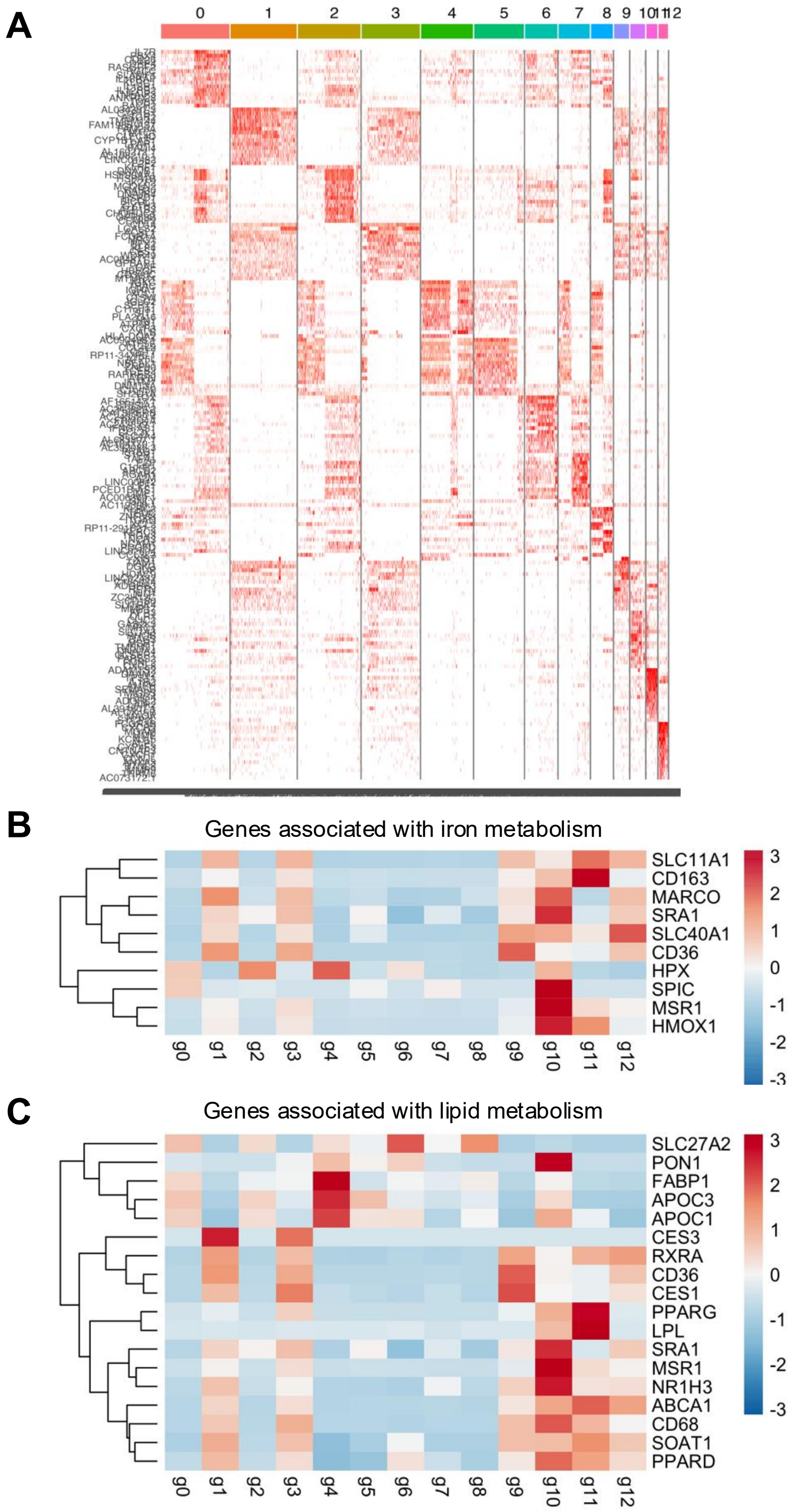

Fig. S2. Single-cell RNA sequencing identifies signature gene expression in healthy controls, AC, and sAH patients. (A) Heatmap depicting the top marker genes defining each macrophage subtype. (B) Heatmap showing the gene expression associated with iron metabolism in each macrophage subtype. (C) Heatmap showing the gene expression associated with lipid metabolism in each macrophage subtype.

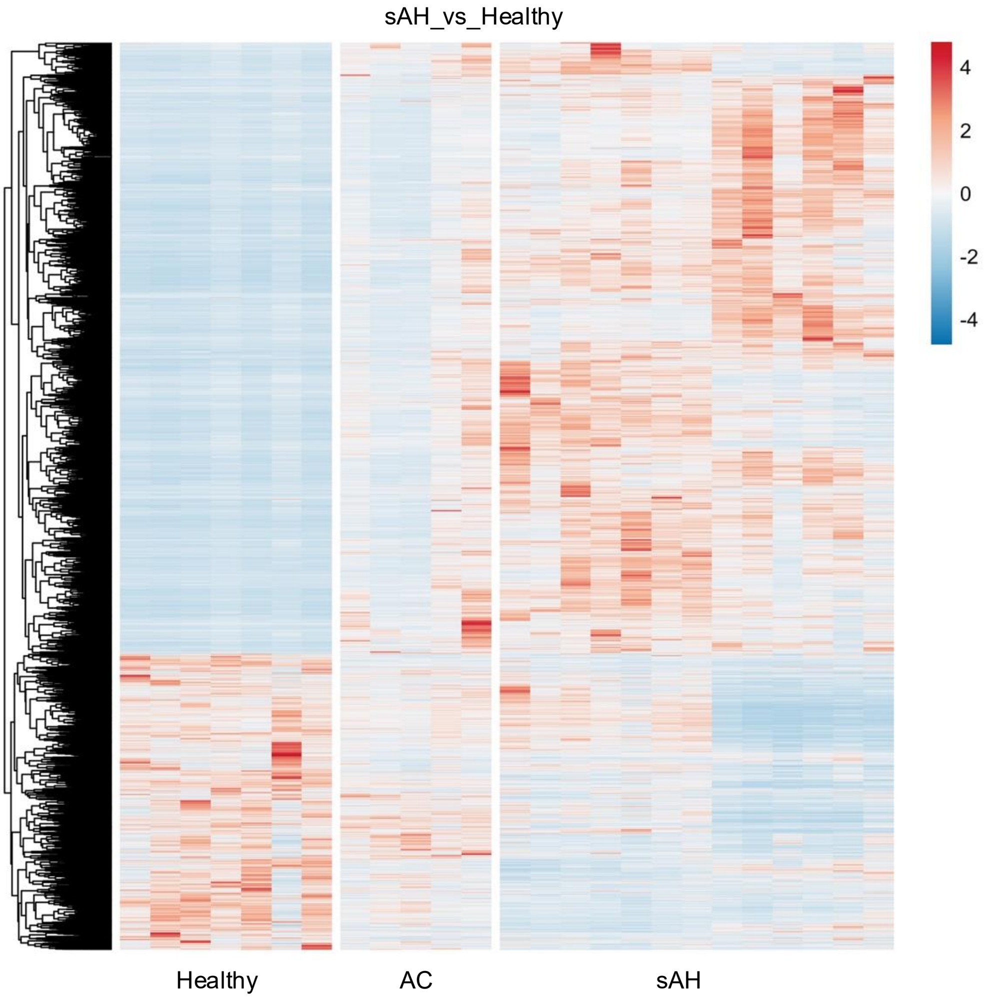

Fig. S3. Heatmap showing differentially expressed genes in the livers of healthy controls and sAH patients.

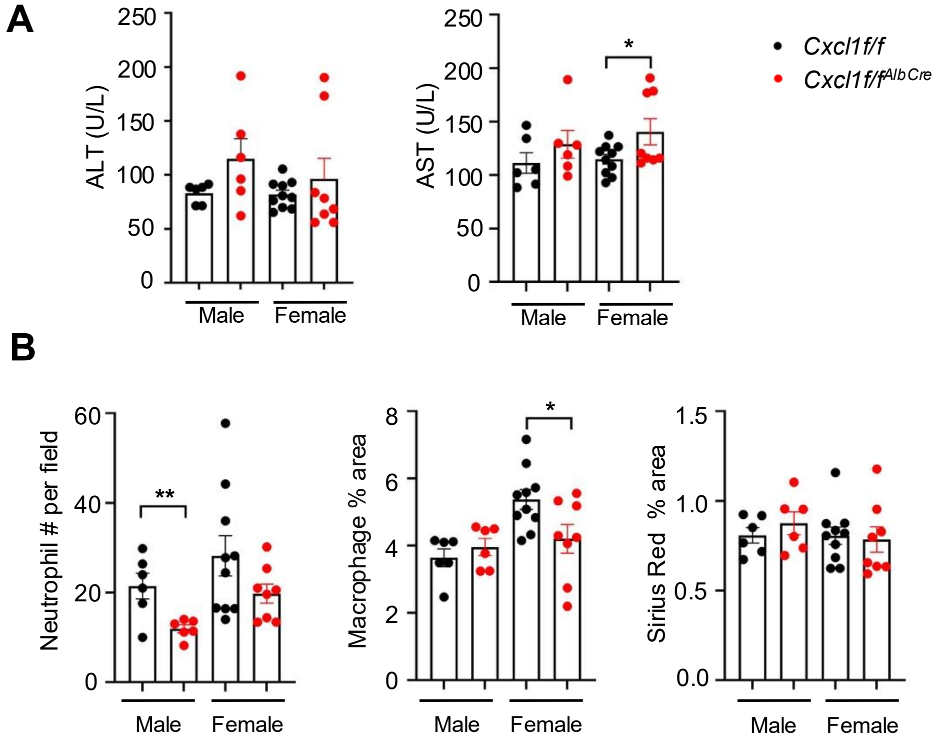

Fig. S4. Comparison of liver injury and neutrophil infiltration in WT and *Cxcl1f/f*^{AlbCre} mice in the EtOH-feeding model. (A) WT mice and *Cxcl1f/f*^{AlbCre} mice were subjected to the chronic-plus-single binge model. Serum ALT and AST levels were measured. (B) Liver tissues were subjected to HE staining, IHC staining of neutrophils (S100A9) and macrophages (F4/80), and Sirius Red staining. Quantification of neutrophils, macrophages, and Sirius Red is shown. Scale bars, 100 µm. Values are represented as mean ± SEM. *$P < 0.05$, as determined by 2-tailed Student's *t* test for comparing 2 groups.

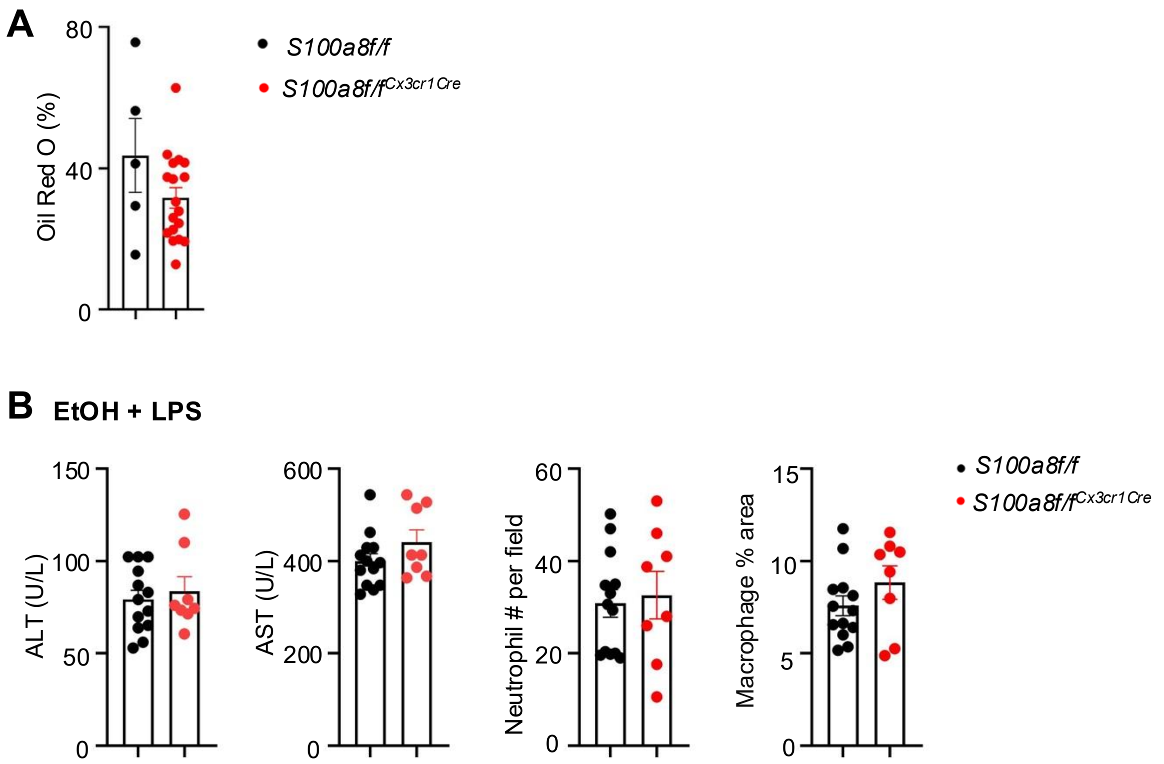

Fig. S5. (A) WT, macrophage-specific *S100a8* KO mice (*S100a8f/f*^{Cx3cr1Cre}) were subjected to the chronic-plus-binge ethanol model. Liver tissues were subjected to Oil Red O staining. Quantification of Oil Red O staining is shown. (B) WT, macrophage-specific *S100a8* KO mice (*S100a8f/f*^{Cx3cr1Cre}) were subjected to the chronic-plus-binge ethanol and LPS model. Serum ALT and AST were measured. Liver tissues were subjected to IHC staining of neutrophils and macrophages. Quantification of neutrophils and macrophages is shown. Values are represented as mean ± SEM. $^*P < 0.05$, as determined by 2-tailed Student's *t* test for comparing 2 groups.

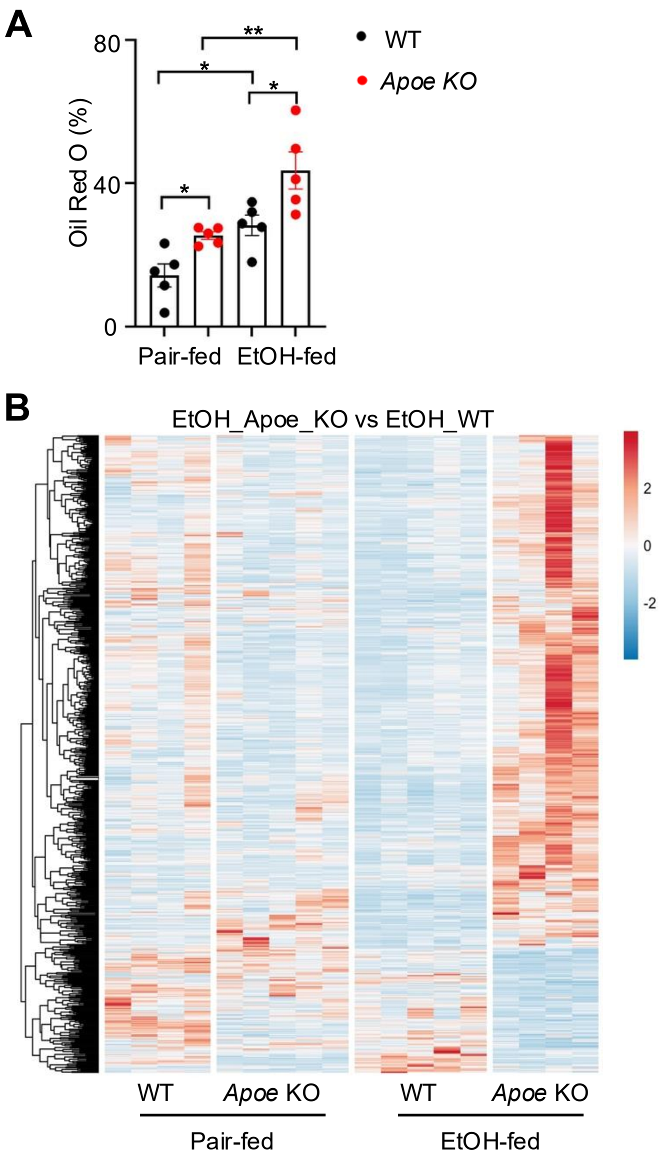

Fig. S6. (A) WT mice and *Apoe* KO mice were subjected to the chronic-plus-single binge model. Liver tissues were subjected to Oil Red O staining. Quantification of Oil Red O staining is shown. Values are represented as mean ± SEM. $^{*}P < 0.05$, $^{**}P < 0.01$, as determined by a 2-tailed Student's *t* test for comparing two groups. (B) Heatmap shows differentially expressed genes in the livers of EtOH-fed WT and *Apoe* KO mice.
